# Direct Surface Patterning of Microscale Well and Canal Structures by Photopolymerization of Liquid Crystals with Structured Light

**DOI:** 10.1021/acsami.2c20739

**Published:** 2023-02-17

**Authors:** Sayuri Hashimoto, Norihisa Akamatsu, Yoshiaki Kobayashi, Kyohei Hisano, Miho Aizawa, Shoichi Kubo, Atsushi Shishido

**Affiliations:** † Laboratory for Chemistry and Life Science, Institute of Innovative Research, Tokyo Institute of Technology, 4259 Nagatsuta, Midori-ku, Yokohama 226-8503, Japan; ‡ Department of Chemical Science and Engineering, Tokyo Institute of Technology, 2-12-1 Ookayama, Meguro-ku, Tokyo 152-8552, Japan; § PRESTO, JST, 4-1-8 Honcho, Kawaguchi 332-0012, Japan; ∥ Living Systems Materialogy (LiSM) Research Group, International Research Frontiers Initiative (IRFI), Tokyo Institute of Technology, 4259 Nagatsuta, Midori-ku, Yokohama 226-8501, Japan

**Keywords:** liquid crystals, coatings, photopolymerization, surface topography, molecular diffusion, structured light, photoalignment

## Abstract

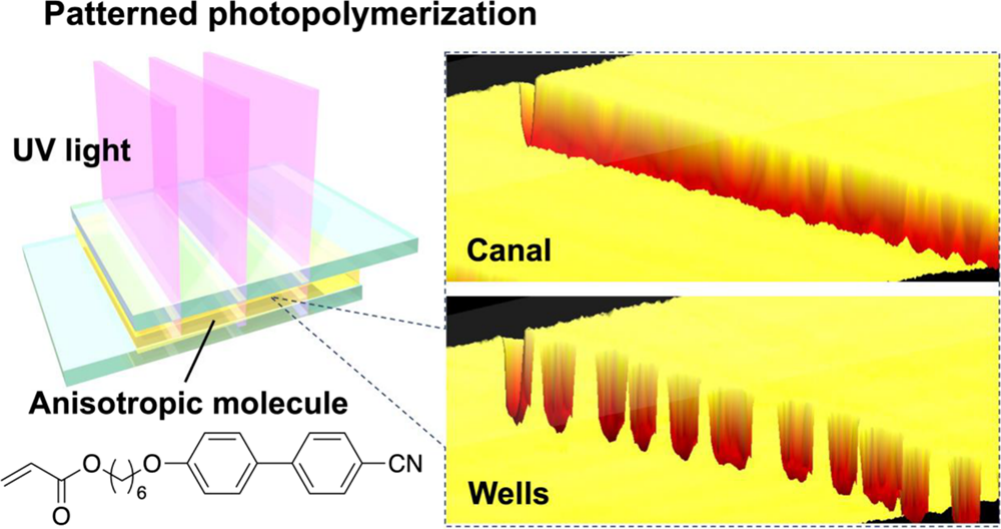

Precise control of the surface topographies of polymer materials is key to developing high-performance materials and devices for a wide variety of applications, such as optical displays, micro/nanofabrication, photonic devices, and microscale actuators. In particular, photocontrolled polymer surfaces, such as photoinduced surface relief, have been extensively studied mainly through photochemical mass transport. In this study, we propose a novel method triggering the mass transport by photopolymerization of liquid crystals with structured light and demonstrate the direct formation of microscale well and canal structures on the surface of polymer films. The wells and canals with depths of several micrometers and high aspect ratios, which are 10 times larger than those of previously reported structures, were found to be aligned in the center of non-irradiated areas. Furthermore, such well and canal structures can be arranged in two dimensions by designing light patterns. Real-time observations of canal structure formation reveal that anisotropic molecular diffusion during photopolymerization leads to a directed molecular alignment and subsequent surface structure formation. We believe that our proposed approach to designing microscale surface topographies has promising applications in advanced optical and mechanical devices.

## Introduction

Surface topography plays an important role in yielding high functionality such as tuning wettability and adhesiveness, modulating optical diffraction and antireflection behaviors, and providing templates for microfabrication.^1−3^ In particular, ordered surface topography of soft materials, represented by polymers and liquid crystals (LCs), provides excellent flexibility and stimuli responsiveness, which is useful for designing next-generation materials in the fields of soft robotics and wearable/flexible electronics.^4−7^ Among various control methods, photocontrol of surface topography has attracted much attention due to its non-contact process and high spatial resolution. For the past decades, the control of surface topographies has achieved impressive success based on photochemical mass transport including polymerization-induced diffusion,^8−11^ phase transition induced by trans/cis photoisomerization,^12−16^ photocrosslinking,^17,18^ and Marangoni flow.^19,20^ In these cases, surface topographies typically exhibit a sinusoidal shape in coincidence with the light intensity profile that triggers the transport of polymers. The surface structures show dimensions with a height of less than several hundred nanometers and a width of a hundred or some tens of micrometers.^8^ The use of sophisticated optics, such as a holographic technique, has also allowed the formation of surface structures with widths of a few micrometers or less.^21^

Recently, we reported that photopolymerization-induced mass transport can produce surface structures of several hundred nanometers in depth.^22−24^ Furthermore, we found that photopolymerization with scanning slit light causes mutual molecular diffusion between polymers and monomers and that the diffusion is maintained in a steady state by the scanning light, leading to a continuous molecular flow. Of particular interest here is that the molecular flow enables the alignment of polymer main-chain and side-chain mesogens along the flow direction, and the resultant polymer film has a flat surface without any change of surface topographies.^25−30^ This interesting phenomenon of photopolymerization induced by molecular diffusion has the potential for creating novel surface topography; however, its detailed investigation remains unexplored.

Here, we report the spontaneous formation of well- and canal-like microscale concave structures by irradiating with two-dimensional (2D) light patterns. Photopolymerization of a monomer directly generated canal, well, and random structures with a dimension of a depth of ∼1.5 μm and a width of 5–10 μm on the surface of polymer films, which shows a high aspect ratio on the order of 10^–1^. Real-time observations of the photopolymerization process were performed to investigate the formation mechanism of the canals, wells, and random structures. Moreover, we found that microscale canals and wells were arranged in a circular and periodic manner simply by irradiation with a circular light pattern. This method of controlling surface topographies, which does not require interference exposure or photoresponsive molecules, will open a pathway to form a variety of complex surface structures through a simple design of structured light.

## Results and Discussion

### Photopolymerization-Induced Surface Topographies

**Figure 1:**
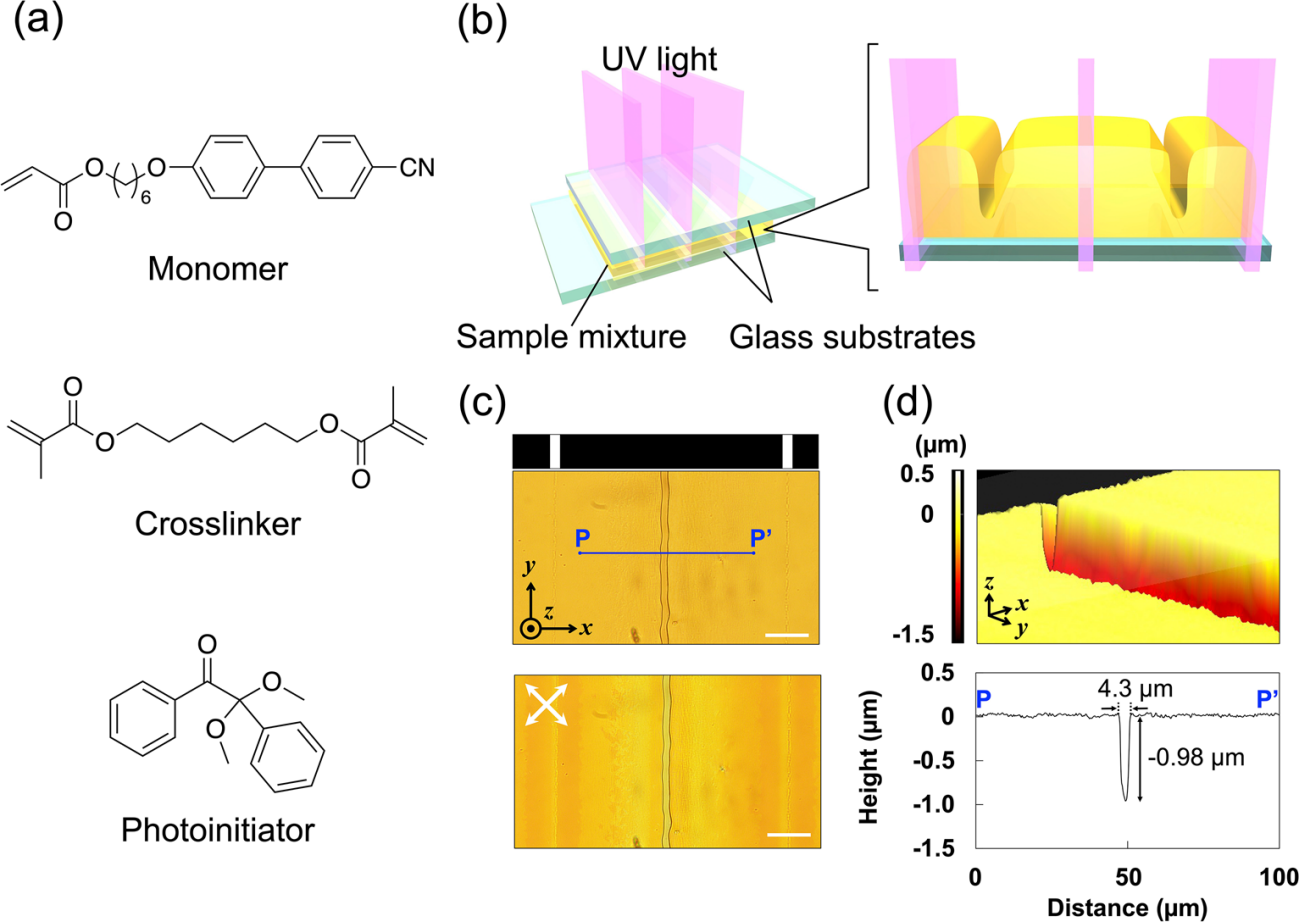
(a) Chemical structures of the materials used in this study. (b) Schematic representation of the patterned photopolymerization process. Surface structures with depths of several micrometers are formed in the center of non-irradiated areas. The thickness of the glass cell is 2–3 μm. (c) Polarized optical micrographs of the films observed under open nicols (upper) and crossed nicols. The film was photopolymerized with an irradiation width of 5.2 μm and a dark-stripe width of 104 μm. The white and black stripes denote the irradiated light pattern. The scale bars are 20 μm. The crossed white arrows denote the polarizer directions. The black arrows and circle represent coordinate axes. (d) 3D surface topography image and cross-sectional view of the film surface profiles in the region marked *P*–*P*′ observed with a confocal laser microscope. The black arrows show coordinate axes.

A sample mixture of a monomer, a crosslinker, and a photoinitiator was employed to fabricate controlled surface topography in LC polymer films (Figure 1a and see also the Materials and Methods section and Figure S1). To form a canal-shaped surface structure, photopolymerization of the mixture was carried out in the glass cell at 100 °C by stripe-patterned ultraviolet (UV) light irradiation with a UV digital light processor (UV-DLP), as shown in Figure 1b (λ = 369 nm; light intensity, 10 mW/cm^2^; irradiation time, 3 min). Here, the UV-DLP produced the widths of the irradiated (white-stripe) and non-irradiated (dark-stripe) areas of 5.2 and 104 μm, respectively. Observations of the photopolymerized film with a polarized optical microscope (POM) under both open and crossed nicols showed periodic line textures in the center of non-irradiated areas, which suggests the formation of defects and/or surface structures (Figure 1c). The top side of the glass substrate was removed, and the surface of the film was observed with a confocal laser microscope. As shown in Figure 1d, these linear textures were canal structures with a depth of 0.98 μm and a width of 4.3 μm. The canal structure has an average aspect ratio (depth-to-width ratio) of 0.19 ± 0.055, which is an order of magnitude higher than the value of 10^–2^ previously reported in conventional mass transport systems induced by photoisomerization and photopolymerization.^10,12,15^ Additionally, patterned photopolymerization of the mixture was conducted at 80, 120, and 150 °C. Such large depths in the canal structures were obtained at the polymerization temperatures below 100 °C (Figure S2).

**Figure 2:**
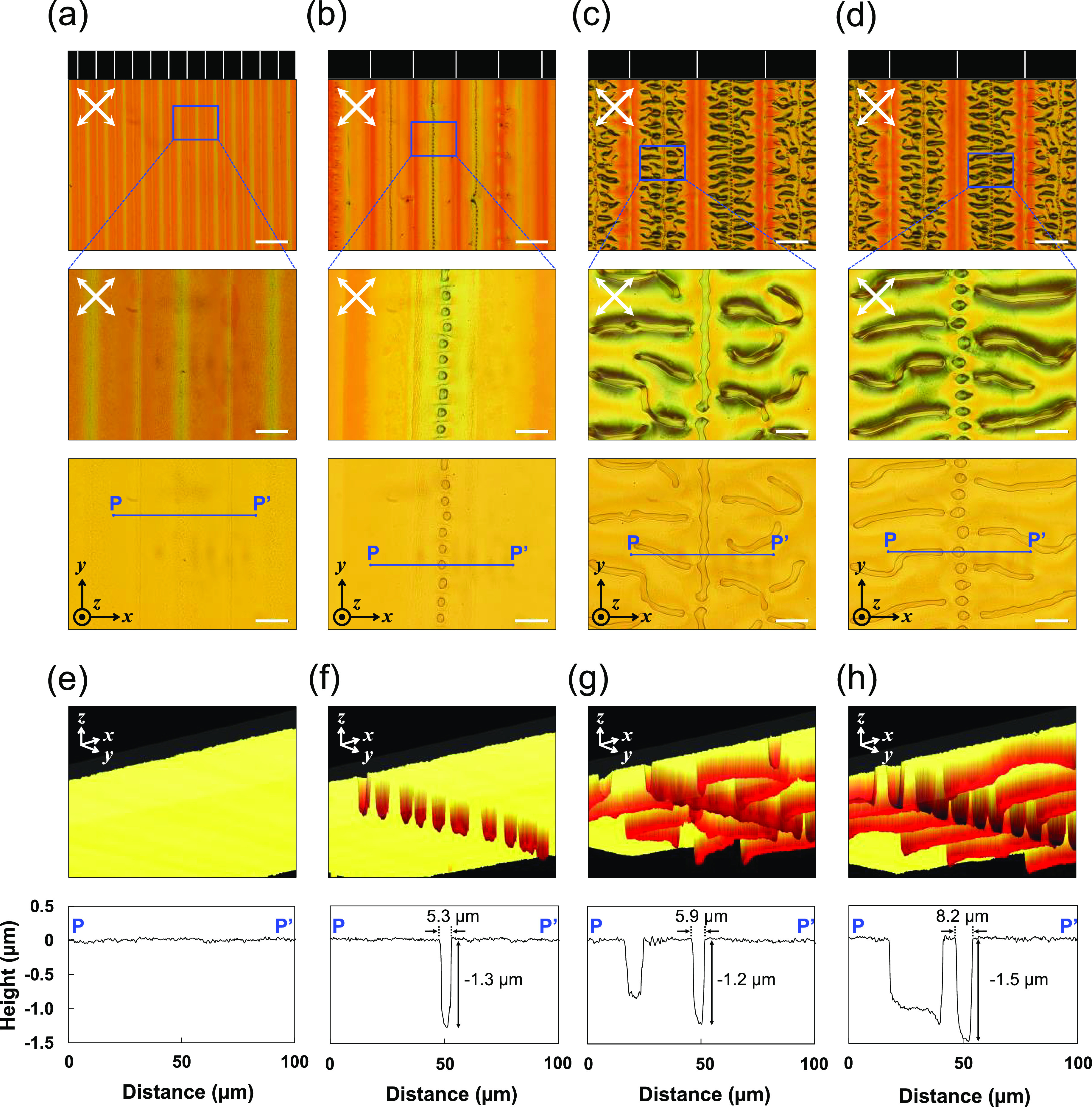
(a–d) POM images of the photopolymerized films observed under crossed nicols (upper and middle) and open nicols (lower). The scale bars are 100 μm (upper) and 20 μm (middle and lower). The areas surrounded by blue squares in the upper images are magnified in the middle and lower images. The dark-stripe widths are (a) 52 μm, (b) 130 μm, and (c and d) 209 μm. Various surface structures are formed: (b) the periodically aligned wells, (c) canals and random structures, and (d) wells and random structures. The white and dark stripes denote the irradiated light pattern. The crossed white arrows show the polarizer direction. The black arrows and circle represent coordinate axes. (e–h) 3D surface topography images (upper) and cross-sectional views (lower) of the film surface profiles in the region marked *P*–*P*′ observed with a confocal laser microscope: (e) flat surface, (f) wells, (g) canals and random structures, and (h) wells and random structures. The white arrows show coordinate axes.

We have explored the effect of stripe patterns during photopolymerization on the manifestation of surface structures; the irradiation width was maintained at 5.2 μm, and the dark-stripe width was varied from 52 to 209 μm. As shown in Figure 2, the widths of the dark stripe greatly affected the shape of the surface structures. More specifically, as the dark-stripe width increased, the surface topography became more complex, and the depth of the surface structures was larger. Although the polymer film obtained using the dark-stripe width of 104 μm showed a canal-like surface (Figure 1), the film irradiated with the narrowest dark stripe of 52 μm formed a flat surface with a defect line in the center of non-irradiated areas (Figure 2a). It is noteworthy that the film exhibited microscale well-like structures on the surface (well depth = 1.3 μm) when photopolymerized with the light pattern with a dark-stripe width of 130 μm. In addition, the wells were periodically aligned in the center of non-irradiated areas (Figure 2b). The film polymerized with a dark-stripe width of 209 μm possessed both the canal and/or well structures in the center of the non-irradiated areas and random structures in the non-irradiated areas (Figure 2c,d). They exhibited a maximum depth of ∼1.5 μm (Figure 2h). These results indicate that the formation of various surface structures, including wells, canals, and random structures with high aspect ratios, can be achieved simply by conducting patterned photopolymerization with different dark-stripe widths. As a result of extensive experiments, we confirmed the reproducible formation of canals, wells, and random structures. However, the structure formation behavior seems to be complex; for example, in the condition of well formation, in which the width of non-irradiated areas is 130 μm, canal structures and/or random structures were also formed, as shown in Figure 2b. We consider that these structures can be simultaneously produced in the non-equilibrium state. Variation of the depth was further investigated in terms of the polymerization temperature (Figure S2). The large depth of canal structures was maintained regardless of the polymerization temperature, showing low standard deviations. The width of the non-irradiated area most strongly affects which structures are preferentially formed.

### Real-Time Observations of Surface Topography Formation

**Figure 3:**
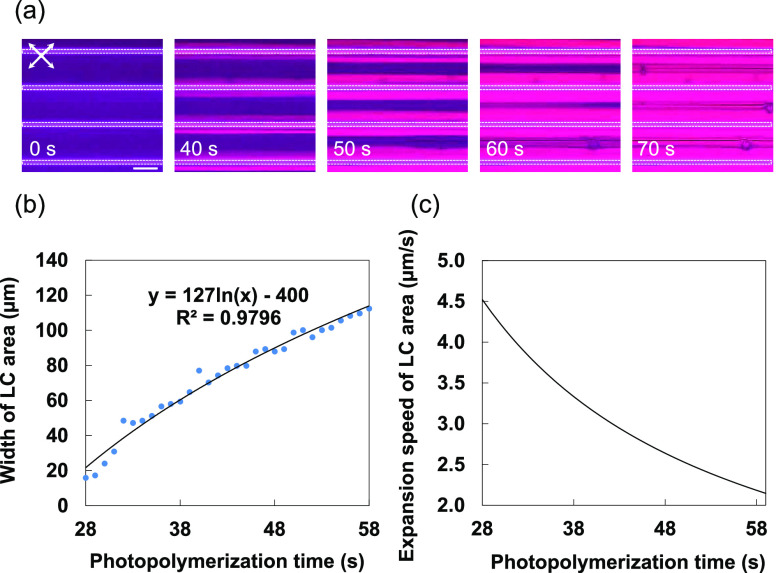
(a) Snapshots of Supplementary Movie 1, which shows the photopolymerization process carried out using the POM–DLP with a white-stripe width of 13 μm and a dark-stripe width of 260 μm, at times of 0, 40, 50, 60, and 70 s. The white dashed lines denote the irradiated areas, and the scale bar is 200 μm. The crossed white arrows represent the polarizer directions. (b) Blue dots show the experimental value of the distance between the boundary line of the LC area and the edge of the irradiated area as a function of the photopolymerization time. The black line shows the approximation of the log function of the experimental value. (c) Expansion speed of the LC area as a function of the photopolymerization time. The LC phase appeared after 28 s of irradiation.

A specially designed POM combined with the DLP system (POM–DLP), which enables the real-time observation of the photopolymerization process, was used to investigate a detailed mechanism of the surface topography formation. The snapshots from Supplementary Movie 1 (see Figure 3a) successfully visualized the process of canal structure formation, where the photopolymerization is carried out with a white-stripe width of 13 μm and a dark-stripe width of 260 μm. After 28 s of irradiation in the white stripe, the irradiated areas became bright. The uniform brightness of the deep pink color indicates that high optical anisotropy emerged in the film and reflects the existence of an LC phase. Note that the monomers used in this study show no LC phase, i.e., no optical anisotropy, whereas the polymers exhibit an LC phase at 100 °C of a photopolymerization temperature (Figure S3). Therefore, the LC phase should appear at a certain polymer concentration as the photopolymerization proceeds during photoirradiation. Fourier transform infrared spectroscopy (FTIR) and POM measurements of the photopolymerized samples irradiated with various light doses revealed that the LC phase appeared when the polymer conversion rate was approximately 60% or more (Figure S4). This clearly indicates that the POM–DLP can visualize the area containing a polymer concentration of >60% as the bright area. The real-time monitoring revealed that the LC area expanded toward the non-irradiated area with the increase in the irradiation time: after 40 s of irradiation, the bright areas began to spread toward the non-irradiated area. When the width of the LC area, which is defined as the distance between the boundary line of the bright area and the edge of the irradiated area, reached about 100 μm after irradiation for 50–60 s, the linear textures were generated in the center of non-irradiated areas. These textures were confirmed by confocal laser microscopy to be canal structures, as shown in Figure 1. These results indicated that the canal structures and LC phase are coincidentally formed during the patterned photopolymerization process.

The formation process of random structures was similarly investigated using the POM–DLP with a white-stripe width of 13 μm and a dark-stripe width of 520 μm, twice the width of the canal formation process (Supplementary Movie 2). Very interestingly, during random structure formation, the width of the LC area reached ∼100 μm as in the case of canal structure formation. The key to generating such surface topographies may lie in the movement of the boundary line of the LC area during photopolymerization.

We successfully formed various surface structures with depths of several micrometers in the center of non-irradiated areas by stripe-patterned photopolymerization. In our previous study, stripe-patterned photopolymerization with the same widths of white and dark stripes produced sinusoidal structures with a height of several hundred nanometers, which was attributed to mutual molecular diffusion between the irradiated and non-irradiated areas.^24^ By contrast, in this study, we use the irradiation pattern where the dark stripe is much wider than the white stripe. The films showed no convex structures in the center of the irradiated areas (i.e., no peaks). Thus, the formation of various surface topographies suggests the existence of another mechanism based on molecular motion between the irradiated and non-irradiated areas.

### Analysis of Expansion Behavior of the LC Area

Figure 3b shows the distance between the boundary line of the LC area and the edge of the irradiated area as a function of the photopolymerization time. The distances were estimated from Supplementary Movie 1 using ImageJ software. The coefficient of determination, *R*^2^, was found to be as high as 0.9796 when fitting the experimental values using the logarithmic function, clearly indicating a logarithmic increase in the LC area. Figure 3c shows the expansion speed of the LC area as a function of time, calculated by differentiating the approximation of the logarithm function. As indicated, the initial speed is ∼4.5 μm/s. The speed is correlated to the molecular diffusion occurring during photopolymerization.^23^ In the photopolymerization process, shear stress is generated by the mutual molecular diffusion between the polymers in the irradiated area and the monomers in the non-irradiated area, forming a unidirectional molecular alignment along the diffusion direction. In fact, POM–DLP observations confirmed that the LC area has a uniform molecular alignment in the direction perpendicular to the border line between the irradiated and non-irradiated areas (Figure S5). Therefore, it can be concluded that the diffusion of polymers into the non-irradiated area and the subsequent increase in the polymer concentration sufficient to exhibit the LC phase lead to an expansion of the LC area during photopolymerization.

As shown in Figure 3c, the expansion speed of the LC area decreases exponentially to ∼2 μm/s when the canals are formed. During the random structure formation, the expansion speed of the LC area was also 2 μm/s, although the initial speed was slightly different. This result suggests that the formation of surface topographies is related to a decrease in molecular diffusion, as shown in the mechanism described below.

**Figure 4:**
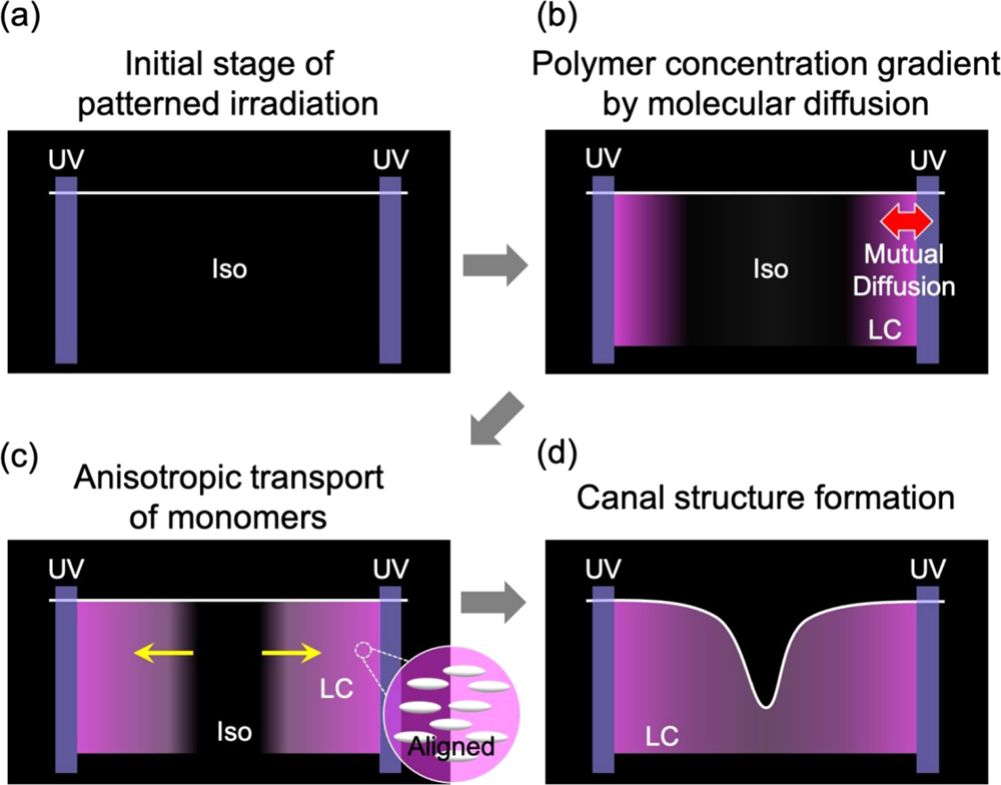
Schematic illustrations of the formation mechanism of canal structures. The pink and black areas represent the LC and isotropic (Iso) areas, respectively. (a) Initial stage of patterned irradiation. (b) Induction of a polymer concentration gradient by molecular diffusion. The red arrow denotes the direction of mutual diffusion between the monomers and the polymers. (c) Anisotropic transport of monomers maintaining their molecular alignment. The yellow arrows represent directed monomer transport into the LC phase. (d) Canal structures are formed in the center of the non-irradiated area.

Figure 4 illustrates the mechanism by which the canal structure is formed on the surface. In the initial stage of photopolymerization, the patterned UV irradiation generates polymers in the irradiated areas and forms the polymer concentration gradient between the irradiated and non-irradiated areas. This gradient causes mutual diffusion; monomers and polymers oppositely migrate into the irradiated and non-irradiated areas, respectively. During photoirradiation, monomers are constantly provided from the non-irradiated areas, and polymers continuously migrate into the non-irradiated areas. The LC phase appears when the polymer concentration exceeds ∼60% and reaches ∼100 μm after a few tens of seconds. This continuous feeding of monomers is reasonable, as polymer conversion is ∼100% in FTIR measurements. Such mutual diffusion of monomers and polymers generates shear stress to unidirectionally align the LC molecules, forming a flat surface. On the other hand, the expansion speed of the LC area exponentially decreases as the photopolymerization proceeds (Figure 3c). Considering the diffusion anisotropy in the LC phase, in which the diffusion coefficient parallel to the molecular alignment direction is higher than the perpendicular one,^31^ monomers tend to diffuse into the LC area at an accelerated rate, maintaining its parallel alignment. Such a large difference of the movement speed between monomers and polymers results in the formation of canal structures at the end of photopolymerization. Due to the difference of the movement speed between monomers and polymers, the density around the boundary of the LC area might increase, leading to density instability and convection. In fact, the edge of the wells showed a concentric molecular alignment, implying that the concentric molecular flow occurs along the molecular alignment direction (Figure S6). Irradiation with wider non-irradiated areas (i.e., well formation condition) might cause a convection flow in addition to the unidirectional diffusion in the center of the non-irradiated areas where the concentrations of the polymers and monomers are lower and higher than irradiation with narrower non-irradiated areas (i.e., canal formation condition), respectively. Depending on the width of the non-irradiated area, which affects the degree of instability, various surface structures such as canals, wells, and random structures might be formed.

### Two-Dimensional Patterning of Microscale Wells/Canals

**Figure 5:**
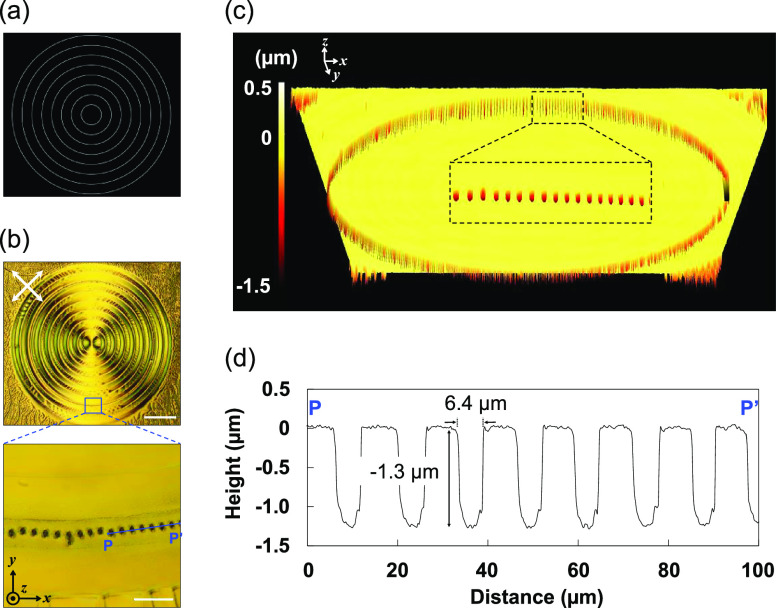
(a) Circular light pattern with a white-stripe width of 5.2 μm and a dark-stripe width of 157 μm. (b) POM images of the obtained film with wells on its surface. The scale bars are 500 μm (upper) and 50 μm (lower). The crossed white arrows represent the polarizer directions, and the blue line denotes the cross-sectional line. The black arrows and circle represent coordinate axes. (c) 3D surface topography image of the film observed with a confocal laser microscope. The white arrows show coordinate axes. (d) Cross-sectional view of the film surface profile measured in the region marked *P*–*P*′ in (b).

As described above, the canals and wells were formed depending on the width of the non-irradiated area. We demonstrate that the surface topography can be designed by the irradiation pattern. Figure 5a shows a circular pattern with a white-stripe width of 5.2 μm and a dark-stripe width of 157 μm. Photopolymerization with this pattern resulted in the periodic formation of microscale wells and/or canals along the irradiation pattern, as can be seen in Figure 5b,d. Each well had a width of 6.4 μm and a depth of 1.3 μm, which are the same dimensions as the wells formed by stripe-patterned irradiation. Furthermore, we found >420 wells regularly arranged at intervals of 13 μm (Figures 5c and S7). This is the first example of the direct formation of high-aspect-ratio microscale canal/well structures via photopolymerization. This method does not require photoresponsive molecules or interference exposures and allows for large-area patterning of complex surface structures. The unique surface topographies with 2D arrangements have potential applications in actuators such as morphing devices with reversible changes in adhesion that can be controlled, coatings with tunable water repellency, and optical devices. For example, periodically arranged microscale canal and well structures might be utilized to diffract light, modulate birefringence, and convert polarization associated with their complex molecular alignment.

## Conclusions

In summary, surface topography with a variety of structures could be fabricated by applying the photopolymerization process using structured light. Photopolymerization of LCs with designed stripe patterns could form canals, wells, and random structures with depths of several micrometers on the surface of LC polymer films. To the best of our knowledge, this is the first time that such complex, deep surface topographies have been fabricated by a single-step photopolymerization process without the use of photoresponsive molecules and polarized light. Very interestingly, more than 400 wells with a depth of ∼1.5 μm were formed by circularly patterned irradiation. Furthermore, we found that the well structures were periodically arranged in a circular pattern. Direct observation of the photopolymerization process revealed that anisotropic molecular diffusion due to spatial polymer concentration gradients was the main factor in the formation of the surface structures. Further optimization of the photopolymerization conditions (e.g., crosslinker concentration, light intensity, and irradiation pattern) will enable us to design highly complex and precisely controlled surface topographies, which are expected to be applied to novel optical diffraction elements and soft actuators.

## Materials and Methods

### Materials

The chemical structures of the materials used in this study are shown in Figure 1. The photopolymerizable monomer 4′-[6-(acryloyloxy)hexyloxy]-4-cyanobiphenyl (A6CB) was provided by ENEOS Corp. (Japan), while the crosslinker 1,6-bis(methacryloyloxy)hexane (HDDMA) was purchased from Tokyo Chemical Industry Co., Ltd. (Japan). The crosslinker was washed with a 5 wt % aqueous sodium hydroxide solution, dried over anhydrous magnesium sulfate, and purified by filtration. The photoinitiator Irgacure 651 was purchased from Tokyo Chemical Industry Co., Ltd. (Japan).

### Preparation of the Glass Cells

For the preparation of the glass cells, glass substrates with dimensions of 25 mm × 15 mm were ultrasonically cleaned in 2-propanol for 30 min, and the cleaned substrates were treated with a UV–ozone cleaner (NL-UV42, Japan Laser Corp., Japan) for 10 min. Subsequently, silane coupling treatment of the substrate was performed for facile separation of the polymer films from the substrate after photopolymerization. More specifically, the cleaned substrates were immersed in an ethanol solution containing a silane coupler (octadecyltrimethoxysilane, Tokyo Chemical Industry Co., Ltd., Japan) at a concentration of 0.2 wt % at 40 °C for 30 min. After heating the glass substrates at 120 °C for 2 h, we obtained the desired surface-treated glass substrates. The glass substrate treated with the silane coupler was then glued to an untreated glass substrate using 2 μm thick silica spacers (Thermo Scientific, USA) (Figure S1a). The thickness of the prepared cell was measured using UV–vis absorption spectroscopy (V-650ST, JASCO Corp., Japan) and calculated using the Fabry–Perot method.^32^

### Fabrication of the Photopolymerizable Film

As previously described in the literature,^24^ we prepared a sample mixture composed of A6CB and HDDMA at a molar ratio of 97:3 and added Irgacure 651 to the mixture at a concentration of 1.0 mol %. The prepared glass cells were filled with the melted sample mixture via capillary force application at 150 °C, which is above the isotropic phase temperature of the mixture. The cells were then cooled to the photopolymerization temperature of 100 °C (Figure S1b), and photopolymerization was carried out using a 369 nm UV light-emitting diode (LED) equipped with a digital micromirror device (DMD; MLS-DIR-LC65365, ASKA Corp., Japan), which can generate arbitrary light patterns designed using illustration software (Adobe Illustrator) (Figure S1c).^33^ More specifically, the irradiation patterns were designed in the form of white and dark stripes, which corresponded to the irradiated and non-irradiated areas, respectively. Our previous work showed that a change in the distance of molecular diffusion (i.e., the width of the irradiation pattern) could lead to the formation of surface relief structures with longer periods.^24^ We designed a range of irradiation patterns by varying the ratio of the irradiated and non-irradiated areas (i.e., with a constant white-stripe width of 5.2 μm and dark-stripe widths of 52, 104, 130, and 209 μm). The sample in the glass cell was then irradiated using the designed pattern at a light intensity of 10 mW/cm^2^ for 3 min. To polymerize any unreacted monomers in the non-irradiated areas, the whole cell was then irradiated with a 365 nm UV-LED light source (LHPUV365-2501, Iwasaki Electric Co., Ltd., Japan) at a light intensity of 5 mW/cm^2^ for 10 min (Figure S1d). After the photopolymerization process, the glass cell was immersed in liquid nitrogen to permit rapid cooling below the glass transition temperature of the polymer. The surface profiles of the films (after the removal of the silane-treated glass substrate) were observed with a confocal laser microscope (LEXT OLS5000, Olympus Co., Ltd., Japan) using a 50× objective lens at room temperature. In addition, the molecular alignment of the film was investigated with a POM (BX53, Olympus Co., Ltd., Japan), and the alignment direction was determined using tint plates with retardation of 137 and 530 nm (U-TP137, U-TP530, Olympus Co., Ltd., Japan).^34^

### Real-Time Observations of Patterned Photopolymerization

The glass cells used for the real-time observations of the photopolymerization process were handmade by adhering glass substrates (i.e., a 25 mm × 25 mm glass substrate and an 18 mm × 18 mm microscope coverslip glass) using 2 μm thick silica spacers. A POM–DLP was employed as a UV light source, which was equipped with a POM and a DMD containing a 1280 × 720 array of mirrors and a 374 nm UV-LED. Using a 4× objective lens and a beam profiler, the size of each pixel was determined to be 2.6 μm. To cut off the wavelengths containing the UV segments of the POM optical source, a UV cut-off filter with a long-pass filter (R-62, TOSHIBA Corp., Japan) was placed under the polarizer. The sample mixtures were irradiated using the designed stripe patterns with the white-stripe width of 13 μm and dark-stripe widths of 260 and 520 μm at a light intensity of 20 mW/cm^2^ for 2 min.

### Fourier Transform Infrared Spectroscopy (FTIR) Measurements

For the preparation of the cells, calcium fluoride (CaF_2_) substrates with dimensions of 30 mm × 10 mm without any surface treatment adhered with 2 μm thick silica spacers in the same manner as described in the “Preparation of the Glass Cells” section. The CaF_2_ cell thickness was 2–3 μm, as confirmed by UV–vis absorption spectroscopy. The sample films were prepared for photopolymerization by UV irradiation throughout the area of 10 mm × 5.64 mm at a light intensity of 2.5 mW/cm^2^ for a certain irradiation time. After the photopolymerization, the cells were immersed in liquid nitrogen to obtain the polymer sample below the glass transition temperature. Then, the sample was measured by Fourier transform infrared spectroscopy (FT/IR-6100, JASCO Corp., Japan). The FTIR spectra were gathered from 500 to 4000 cm^–1^ with a resolution of 4 cm^–1^. The polymerization conversion of the sample was calculated by the following equation.
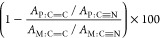
where *A*_*x*:*y*_ (*x* = P for polymer and M for monomer; *y* = bond types) is the absorbance of the stretching vibrations of C=C at 1619–1651 cm^–1^ and that of the stretching vibrations of C≡N at 2197–2234 cm^–1^.^23,30^Figure S4 shows the conversion of the sample films as a function of the exposure dose (= [light intensity]·[irradiation time]). Then, POM observations of all sample cells were performed to determine their LC properties at a temperature of 100 °C.

## Supporting Information Available

Schematics of the preparation of polymer films by patterned photopolymerization; depth of canal structures as a function of the photopolymerization temperature; differential scanning calorimetry thermograms of monomers and polymers; conversion changes of polymer films measured by FTIR; POM images of the polymer film with a canal structure; POM images of the polymer film with well structures; and POM images of the polymer film irradiated with a circular pattern (PDF)
Detailed POM observation of the formation process of the canal structures (MP4)
Detailed POM observation of the formation process of the random structures (MP4)
