# Supplementary material for: Direct Surface Patterning of Microscale Well and Canal Structures by Photopolymerization of Liquid Crystals with Structured Light
Source: ACS Appl Mater Interfaces. 2023 Feb 17;15(11):14760–7. doi: 10.1021/acsami.2c20739 (PMC10037240; doi:10.1021/acsami.2c20739)
Supplement: Supplementary file 1 — am2c20739_si_001.pdf [file am2c20739_si_001.pdf]

Supporting Information

Direct Surface Patterning of Microscale Well and Canal Structures by Photopolymerization of Liquid Crystals with Structured Light

Sayuri Hashimoto,[1,2] Norihisa Akamatsu,[1,2] Yoshiaki Kobayashi,[1,2] Kyohei Hisano,[1,2] Miho Aizawa,[1,2,3] Shoichi Kubo,[1,2] and Atsushi Shishido*[1,2,4]

[1]Laboratory for Chemistry and Life Science, Institute of Innovative Research, Tokyo Institute of Technology, 4259 Nagatsuta, Midori-ku, Yokohama 226-8503, Japan
[2]Department of Chemical Science and Engineering, Tokyo Institute of Technology, 2-12-1 Ookayama, Meguro-ku, Tokyo 152-8552, Japan
[3]PRESTO, JST, 4-1-8 Honcho, Kawaguchi 332-0012, Japan
[4]Living Systems Materialogy (LiSM) Research Group, International Research Frontiers Initiative (IRFI), Tokyo Institute of Technology, 4259 Nagatsuta, Midori-ku, Yokohama 226-8501, Japan
*E-mail: ashishid@res.titech.ac.jp

This file includes:

Figure S1. Schematics of the preparation of polymer films by patterned photopolymerization.

Figure S2. Depth of canal structures as a function of the photopolymerization temperature.

Figure S3. Differential scanning calorimetry thermograms of monomers and polymers.

Figure S4. Conversion changes of polymer films measured by FTIR.

Figure S5. POM images of the polymer film with a canal structure.

Figure S6. POM images of the polymer film with well structures.

Figure S7. POM images of the polymer film irradiated with a circular pattern.

Supplementary movie 1. Detailed POM observation of the formation process of the canal structures.

Supplementary movie 2. Detailed POM observation of the formation process of the random structures.

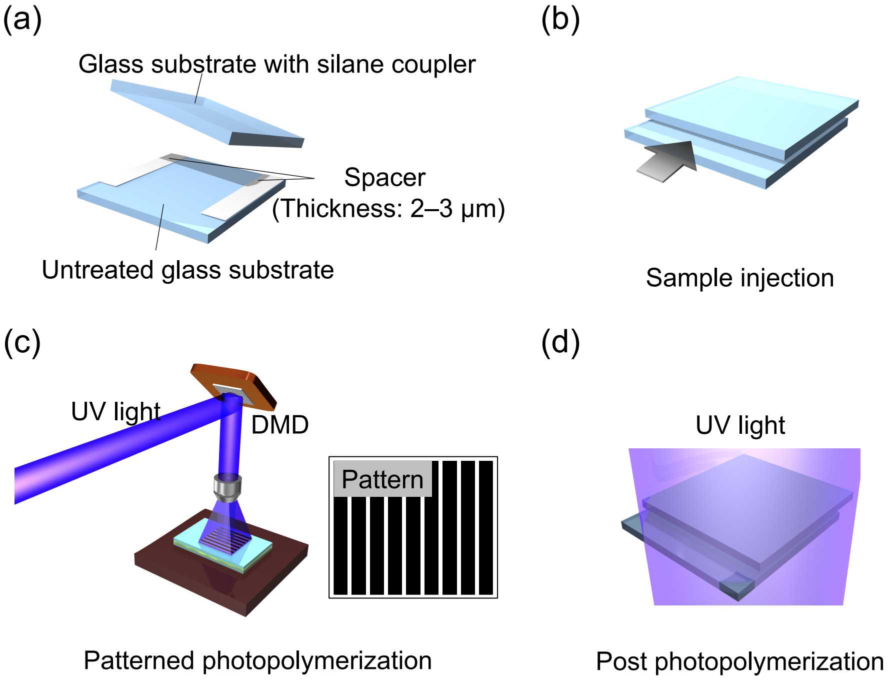

Figure S1. Schematics of the preparation of polymer films by patterned photopolymerization. (a) Fabrication of a glass cell. (b) Sample injection. (c) Patterned photopolymerization of a sample with DMD and image of the irradiation pattern. (d) Post photopolymerization.

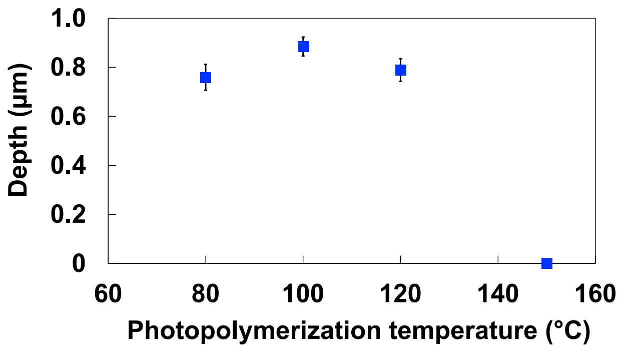

Figure S2. Depth of canal structures as a function of the photopolymerization temperature. All of the films were obtained by photopolymerization with the irradiation width of 5.2 μm and the dark-stripe width of 130 μm at the light intensity of 10 mW/cm^2. Three experiments were performed at each temperature, and error bars indicate standard deviations.

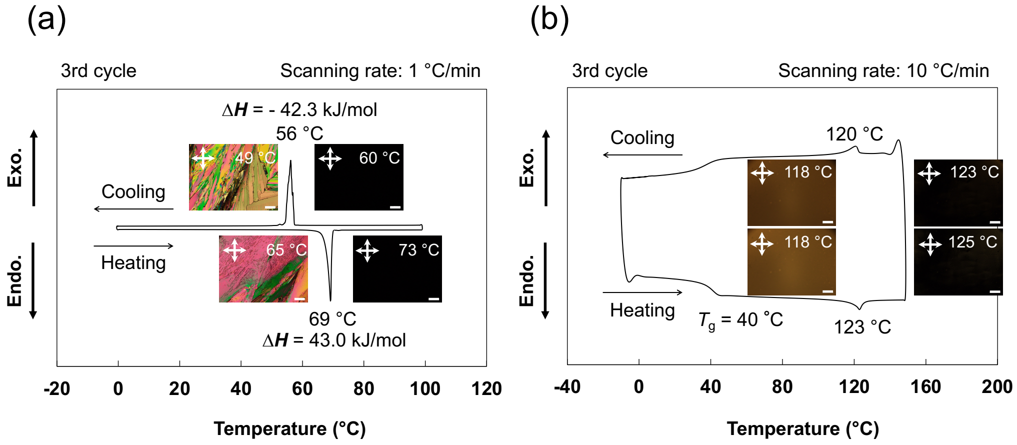

Figure S3. Differential scanning calorimetry thermograms and POM images of monomer (A6CB) (a) and polymer (PA6CB) (b). Crossed arrows show the direction of polarizers. Scale bars, 200 μm.

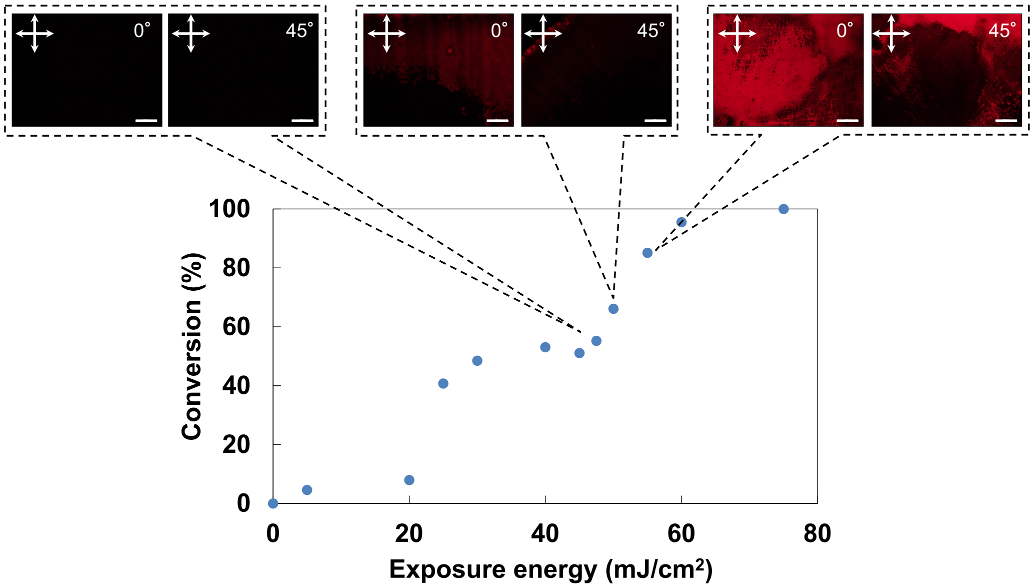

Figure S4. POM images observed at the temperature of 100 °C (upper) and the conversion of polymer films as a function of exposure energy (lower) measured by FTIR. Crossed arrows show the direction of polarizers. Scale bars, 500 μm.

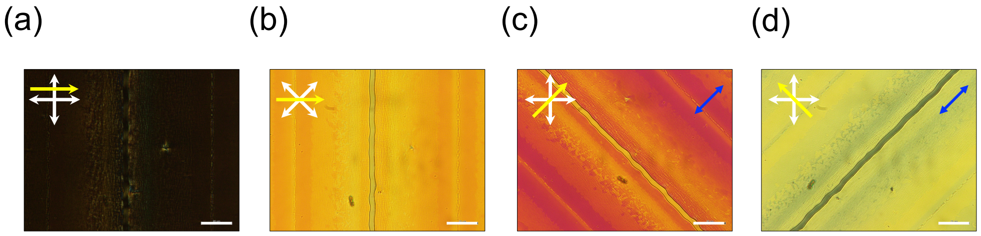

Figure S5. POM images of the polymer film with a canal structure under crossed polarizers rotated at 90° (a, b), 45° (c), and −45° (d) with a tint plate (c, d). Scale bars are 20 μm. White arrows show the direction of polarizers. Yellow arrows denote the lattice vector to the direction of stripe. Blue arrows show the slow axis of the tint plate with a retardation of 137 nm.

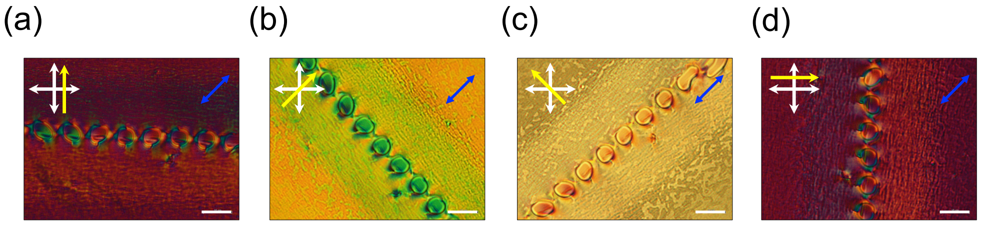

Figure S6. POM images of the polymer film with well structures under crossed polarizers rotated at 0° (a), 45° (b), −45° (c), and 90° (d) with a tint plate. Scale bars are 10 μm. White arrows show the direction of polarizers. Yellow arrows denote the lattice vector to the direction of stripe. Blue arrows show the slow axis of the tint plate with a retardation of 530 nm.

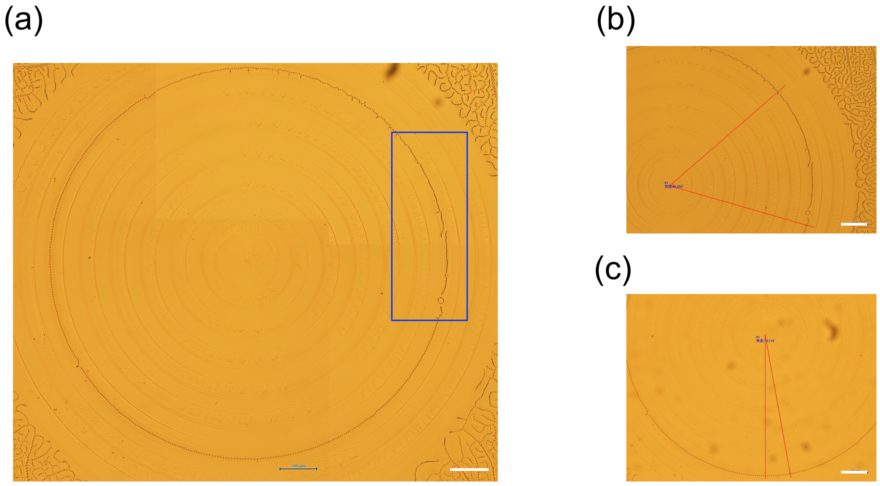

Figure S7. (a) POM images of the film with more than 420 wells and short length of canals observed under open nicols. The film was obtained by photopolymerization using circular pattern. The number of wells was calculated; (b) blue area has 30 wells and canals, (c) the other area has 394 wells or more. Scale bars, 200 μm.

Supplementary Movies Description

Supplementary Movie 1. Detailed POM observation of the formation process of the canal structures. The film was photopolymerized by POM−DLP with irradiation patterns of a white-stripe width of 13 μm and dark-stripe width of 260 μm.

Supplementary Movie 2. Detailed POM observation of the formation process of the random structures. The film was photopolymerized by POM−DLP with irradiation patterns of a white-stripe width of 13 μm and dark-stripe width of 520 μm.
